# Non-Coding RNAs in Human Health and Diseases

**DOI:** 10.3390/genes14071429

**Published:** 2023-07-11

**Authors:** Deborah J. Good

**Affiliations:** Department of Human Nutrition, Foods, and Exercise, Virginia Tech, Blacksburg, VA 24060, USA; goodd@vt.edu; Tel.: +1-540-231-0430

**Keywords:** ncRNA, microRNA, long non-coding RNA, piwi RNA, circular RNA, sno RNA, small non-coding RNA

## Abstract

Non-coding RNAs (ncRNAs) are, arguably, the enigma of the RNA transcriptome. Even though there are more annotated ncRNAs (25,967) compared to mRNAs (19,827), we know far less about each of the genes that produce ncRNA, especially in terms of their regulation, molecular functions, and interactions. Further, we are only beginning to understand the role of differential regulation or function of ncRNAs caused by genetic and epigenetic perturbations, such as single nucleotide variants (SNV), deletions, insertions, and histone/DNA modifications. The 22 papers in this Special Issue describe the emerging roles of ncRNAs in neurological, cardiovascular, immune, and hepatic systems, to name a few, as well as in diseases such as cancer, Prader–Willi Syndrome, cardiac arrhythmias, and diabetes. As we begin to understand the function and regulation of this class of RNAs, strategies targeting ncRNAs could lead to improved therapeutic interventions for some conditions.

## 1. Introduction

In the realm of genetics and molecular biology, the focus has traditionally been on understanding the functions and mechanisms of protein-coding genes, which are responsible for producing the proteins that carry out essential cellular processes. These transcripts are arguably easier to study as once the protein that they code for is identified its function and its interactions can be further elucidated. However, it has become increasingly evident that a significant portion of the genome is transcribed into RNA molecules that do not encode proteins. In fact, there are more annotated genes that code for non-coding RNAs (ncRNAs) (25,967) compared to genes coding for proteins (19,827) according to the latest release of the Ensembl! Human assembly (GRCH38.p13, Ensembl! Release, November 2022), but one could argue that we know far less about the regulation, function, and interactions of the majority of these annotated ncRNAs. Interestingly, there are tens of thousands of ncRNAs that have not yet been annotated, as we will see in each section.

As suggested by their name, ncRNAs are RNA molecules that are not translated into proteins but instead exert their functions directly through their RNA transcript. Many of them are transcribed from regions of the genome that were once considered “junk DNA” or non-functional sequences. Over the past few decades, advances in high-throughput sequencing technologies and bioinformatics have unveiled a diverse array of ncRNAs with distinct structures and functions.

**Figure 1:**
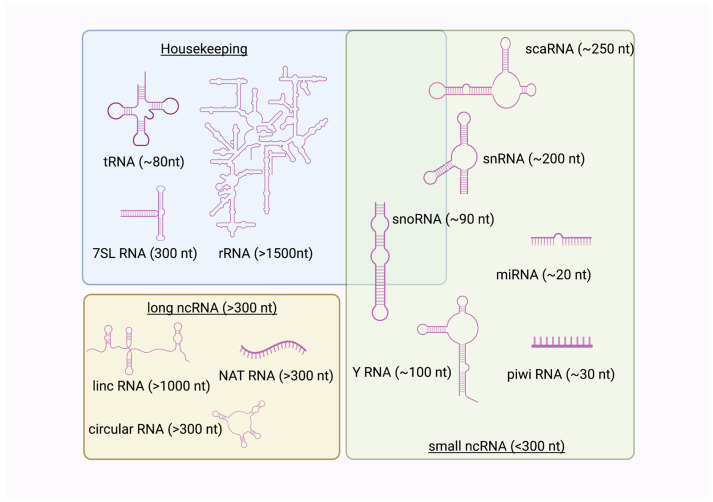
Three classes of non-coding RNAs (ncRNAs). The ncRNAs can be divided into those involved in protein synthesis (housekeeping), and those that participate in RNA and protein regulation, classified by their RNA length. The long and small ncRNA groups have diverse functions, with some snoRNAs and scaRNAs also having housekeeping roles, indicated by the overlap. To date, lncRNAs are implicated in regulation of mRNA synthesis rather than in general housekeeping functions (Created with BioRender.com, accessed on 27 June 2023).

The ncRNAs can be organized into three overlapping groups, based on function (Figure 1) [1,2]. In this review, the housekeeping ncRNAs were organized as a separate grouping as those that perform the basic functions of protein synthesis from RNA, while the small and long ncRNA groupings include those that modulate housekeeping ncRNAs, mRNAs, and other ncRNAs (Figure 1). A recent guide to naming ncRNA genes provides information on all of the major classes of ncRNA [3].

Among the well-studied ncRNAs are the so-called housekeeping ncRNAs, which include transfer RNAs (tRNAs), ribosomal RNAs (rRNAs), and the RNA encoding the signal recognition particle (7SL) [2]. However, as shown in Figure 1, there is overlap here with some members of the small ncRNA family. Some small nucleolar RNAs (snoRNAs), such as the U8 small nucleolar RNA (snoRNA), are involved in ribosomal biogenesis [3,4], while the H/ACA snoRNA involved in ribosomal modification [3]. Cajal body snoRNAs and scaRNAs methylate tRNAs [5,6] and several small nuclear RNAs (snRNA) are part of the large ribonuclear protein spliceosome complex, including U1, U2, U3, U4, and U5, among others [7,8]. Further, the U7 small nuclear RNA (snRNA) mediates pre-mRNA processing of histone RNA as part of the U7 ribonuclear particle [9]. In this Special Issue, there were no published articles on any of the U-family of snoRNAs or snRNAs.

While together tRNAs and rRNAs make up the majority of ncRNAs found in a typical mammalian cell [2], only one of the submitted articles in this Special Issue addressed this group of ncRNAs. Specifically, Qin and colleagues analyzed tRNA-derived small RNAs (tsRNAs), which, as the name suggests, are cleaved from the longer tRNAs to produced small RNA fragments of ~20 nucleotides [10]. This group screened for ncRNAs in muscle-invasive bladder cancer, which accounts for up to 20% of all bladder cancer cases in China. Tumor and control mucosal tissues were obtained from eight patients, with four sets of samples eventually being used for the initial analysis. The group found differential expression of 574 tsRNAs, and 3 tsRNAs in particular whose upregulation (2) or downregulation (1) were correlated with the tumor tissue in the eight patients [10]. This work sets the stage for future studies examining the specific roles of these three tsRNAs in normal and tumor conditions.

The two additional subclasses of ncRNAs, long non-coding RNAs (lncRNA) and small non-coding RNA (sncRNA), are generally less well studied, but we find examples of each subclass in multiple submitted articles for this Special Issue. This Special Issue on “Non-coding RNAs in Human Health and Diseases” has collected 22 independent articles on ncRNAs in various human conditions, including diabetes, obesity, cancer, liver disease, cardiovascular disease, and neurodevelopmental disease. In addition, several articles are focused on methodologies for screening and characterizing these ncRNAs. This editorial will summarize the findings for these 22 articles and place the findings within the larger context of ncRNA biology.

## 2. Small Non-Coding RNAs

The small non-coding RNA family (sncRNA) includes microRNAs (miRNAs), snoRNAs, scaRNAs, Y-RNAs, snoRNAs, and piwiRNAS. This ncRNA family is not classified by function per se but rather by size, with each of the RNA species in this subclass generally having fewer than 300 nucleotides. Y- and scaRNAs were not included as the topic of any of the published articles in this Special Issue, but it is important to highlight them in this context. Y-RNAs have been known about for more than 40 years, but their functional role is still emerging. To date, 966 Y-RNA pseudogenes and 878 predicted Y-RNA transcripts have been identified in humans [11]. This family of ncRNAs interacts with and scaffolds proteins, yielding ribonucleoproteins with enzymatic activity in DNA replication, RNA quality control, transport of RNA to subcellular locations, and response to cellular stress [11]. Small Cajal body RNAs (scaRNAs) are slightly larger than the Y-RNAs, but also function in biogenesis of ribonuclear proteins. They are a subset of the small nucleolar RNA subclass, but contain a CAB motif, which is a long GU nucleotide repeat, which signals for them to be transported to the Cajal body [12]. While not entirely characterized, the Cajal body is a distinct area of the nucleolus that appears to be important for formation of ribonuclear proteins, splicing, ribosome formation, and maintenance of telomeres. Altered levels of Y-RNAs and scaRNA are associated human cancers, and UV stress [12], but more work is needed to understand their roles in the etiology or pathogenesis of other human diseases.

One paper in this Special Issue discussed the unique class of ncRNAs called piwiRNAs. This name is derived from their longer name, P-element-induced wimpy testis (Piwi)-interacting RNAs. These piwiRNAs are slightly larger than miRNA (24–32 nucleotides) and interact with specific PIWI proteins, which, like miRNA-interacting proteins, are members of the Argonaute protein family [13]. Transposable element transcripts are key targets of the PIWI–piwiRNA complex, but the mRNA from protein-coding genes has also been shown to produce piwiRNAs from the 3′untranslated regions, resulting in fine-tuned protein synthesis for that transcript [13]. In the article by Sabbah and colleagues, piRNA-823 was studied as a diagnostic biomarker for colorectal cancer [14]. According to the piwiRNA database, piRNAdb (https://www.pirnadb.org/, accessed on 6 June 2023), piRNA-823, also called hsa-piR-823, along with six other aliases, is formed from the 3′ untranslated region of the *GRK7* gene. The protein product of *GRK7* is a guanine (G-protein) coupled receptor kinase that, according to the GeneCards database (https://www.genecards.org) [15], is expressed in retinal cells, and phosphorylated cone opsins in vision. The GTEX gene expression portal (http://www.gtexportal.org, accessed on 6 June 2024) for GRK7 indicates low overall expression in multiple tissues, including the colon. That brings us back to the article in this Special Issue on piRNA-823, which compared serum levels of piRNA-823 in 84 colorectal cancer patients, compared to healthy controls [14]. They found significantly higher serum levels of piRNA-823 in cancer patients, compared to controls, with a significant correlation with tumor stage, tumor differentiation, and lymph node metastasis. Others have followed up on Sabbah’s work, most recently an article which developed a nanotechnology-based detection platform for measuring piRNA-823 in clinical samples [16].

Together, these small ncRNAs appear to be of emerging importance in the biology of human diseases, especially cancers. As we will see in the next section, the best characterized type of small ncRNA are the microRNAs, and, thus, they deserved their own section in this review. miRNAs are also dysregulated in cancer and other human diseases, and the 10 papers on this family of ncRNAs cover the wide spectrum of their roles.

## 3. MicroRNAs (miRNAs)

MicroRNAs (miRNAs) are prominent members of the ncRNA family, and generally well-studied compared to other sncRNA family members. The miRNA database, miRbase [17] has (as of June 2023) 38,589 entries, covering miRNA species from 271 different mammalian and non-mammalian organisms. The number of entries for *Homo sapiens* is 1917, while there are 1234 entries for *Mus musculus*. Generally, miRNAs fall into two functional categories—those that regulate mRNA stability, and those that regulate RNA translation. As we will see from the 10 articles that focus on miRNAs in this Special Issue, authors discussed these roles within the context of cancer, diabetes, and neurodevelopmental disorders.

**Figure 2:**
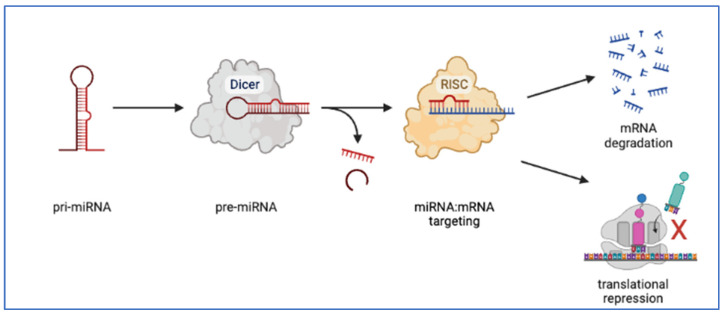
MicroRNA processing and function. Primary miRNA (pri-miRNA) is transcribed and folds into a stem-loop structure. The pre-miRNA is then exported to the cytoplasm where it is cleaved by Dicer endonuclease. Upon cleavage and unwinding by an RNA helicase (not shown), the mature miRNA finds its mRNA targets and in conjunction with an RNA induced silencing complex (RISC) the complex confers either translational repression or mRNA degradation (Created with BioRender.com, accessed on 25 June 2023).

Before discussing the 10 articles on miRNA in this Special Issue, it is important to expand a bit on the role of miRNAs in mRNA regulation. As shown in Figure 2, following transcription and processing, the miRNA finds its target mRNAs and binds to the complementary sequence which is usually found on the 3′-untranslated region (3′UTR) of the mRNA [18]. However, only 10% of cellular miRNA is associated with RISC at a given time and is considered the functional pool [18]. Most articles do not specifically measure the RISC-associated pool when changes in miRNA levels are reported, so it is not always clear how these total miRNA levels will correlate with functional levels of miRNA that are actively inhibiting mRNA translation.

Overall, 5 of the 10 miRNA-focused articles in this Special Issue described the role of miRNAs and their target genes in various types of cancers. Tian and colleagues described a global analysis of miRNA binding sites within the mRNAs of oncogenes and tumor suppressor genes [19]. They used a dataset from the TargetScan miRNA database containing 116,371 predicted miRNA binding sites within 12,436 genes, a dataset from an RNA seq analysis of total polyA RNA in normal versus Dicer1 knockout mouse embryonic stem cells, and an RNA-seq database of RNA polysomes from normal and Dicer1 knockout HCT116 (human colorectal carcinoma cells) to sort “cancer genes”—including oncogenes and tumor suppressor genes into a list containing predicted miRNA binding sites among 733 cancer genes. These genes were then examined using the RNA-seq normal versus Dicer1 knockout datasets. Interestingly, when comparing cancer genes to non-cancer genes, Tian and colleagues found more miRNA binding sites overall in the cancer genes, with many cancer genes having more than 20 conserved miRNA binding sites. Tumor suppressor mRNAs tended to have more miRNA binding sites than oncogenes, suggesting that miRNA targeting of these mRNAs could lead to a lack of growth suppression. However, their results were not able to differentiate between miRNA action on mRNA degradation or translational inhibition and are in opposition to the overall miRNA downregulation patterns seen in many cancers. Indeed, the review by Davies and colleagues on miRNAs in ovarian cancer names several miRNAs that are downregulated in this cancer, including miR-101, miR-584, and miR-27b-5p, and suggests that these can be used as biomarkers for diagnosis and prognosis of these cancers [20]. Alternatively, Setlai and colleagues describe several miRNAs whose expression appears to promote the transition to glioblastoma [21]. For example, miR-451, whose expression is usually downregulated in other cancers, was described as being associated with proliferation, increased metastatic potential, and suppression of the mTOR pathway in glioma. Several other miRNAs whose expression promoted glioblastoma progression included those targeting the tumor suppressor gene PTEN, namely miR-17-5p, miR-23a-3p, and miR-26a-5p, as well as miR-10b-5p that targets the p53 tumor suppression gene [21]. In a research paper by Murakami and colleagues, the downregulation of tumor suppressor miRNAs, miR-34a and miR-34b, and the p53 tumor suppressor gene was correlated with increased MYC oncogene expression in multiple myeloma patients [22]. Finally, Ramorola and colleagues characterized 32 miRNAs associated with HIV-1 exposed Burkitt-lymphoma cells, finding that there was, essentially, an even distribution between those that were downregulated and those that were upregulated (16 each) [23]. However, the exposure of Burkett’s lymphomas cells to HIV-1 lead to the downregulation of miRNAs, such as miR-200c-3p, which has been shown by this group and others to have a tumor suppressor role. Taken together, these five papers demonstrate that the apparent downregulation of miRNAs in cancers may reduce tumor suppressor pathways, leading to more proliferation and overall aggressiveness of the tumors.

The remaining five miRNA papers in this Special Issue focus on other human disease states. Roy and colleagues reviewed miRNA expression in various neurodevelopmental conditions, including Alzheimer’s Disease, Parkinson’s Disease, and Huntington’s Disease, among others [24]. They discuss the use of plasma, cerebral–spinal fluid, or serum miRNA biomarkers for these diseases as future diagnostic tools, which relate directly to the silicon-on-insulator article by Ivanov and colleague who show that differential plasma miRNA expression patterns in autism spectrum disorder could be accurately detected using a silicon nanowire-based nanosensor with complementary to oligonucleotide probes [25]. This unique methodology was able to discriminate between the plasma of patients and control samples, for increases in previously described autism-associated miRNAs, including miR-106a-5p, miR-106b-5p, and miR-494-5p. There currently is not a blood-based test for autism spectrum disorder diagnosis, but this technology has diagnostic potential for autism [25], as well as some cancers tested by the same group (e.g., [26,27,28]). Three papers in this section take an interesting approach of examining variants in miRNAs that may be associated with two diseases—type 2 diabetes mellitus (T2D), and coronary artery disease. Khan and colleagues examined previously characterized single nucleotides variants in miR-196-a2, miR-146a, and miR-423 with their association in T2D patients from the Pakistani population [29]. By using the tetra-primer amplification refractory mutation system “ARMS”-based PCR, variants in two of these miRNAs were shown to have a strong association with T2D in this population. Furthermore, Khan and colleagues studied changes to the predicted secondary structure of miRNAs, using in silico techniques for the normal and variant sequences. A second research study on the Pakistani population looked at polymorphisms associated with cardiovascular disease, which led to up to 17.9 million deaths in 2019, according to the World Health Organization [30]. Haq and colleagues used 223 patients, and 150 controls to find that variants in miR-27A and miR-196-a2 (rs895819, and rs11614913, respectively) showed co-dominant or dominant association with coronary artery disease [31]. Of note, miR-196-a2 was described by the previous study on diabetes [29], and recently also as a marker of colorectal cancer [32], all noting the correlation with rs11614913. Novak and colleagues took a slightly different approach by examining a variant within the putative binding site for miR-31/-584 in the angiotensinogen (AGT) gene, and found that the CC genotype for rs7079 in the 3′ untranslated region of the AGT gene was significantly correlated with earlier onset of clinical referral in patients with coronary artery disease, and presentation with reduced coronary artery diameter (restenosis), after angioplasty [33]. Others had previously shown that the C-allele more strongly associated with both miRNAs, compared to the A-allele, leading to a shift towards reduced AGT expression in carriers [34].

The 10 articles that focused on miRNA regulation in cancer, diabetes, neurodevelopmental conditions, and coronary artery disease present a dynamic picture of differential miRNA levels in patients versus controls that correlate with disease state, but also a role for single nucleotide polymorphisms to affect both target and miRNA interactions and ultimately expression levels. Continued examination of polymorphisms and RNA levels in these disease states should reveal targets both for diagnostic and therapeutic interventions.

## 4. Long Non-Coding RNAs

Since the original description of the XIST long non-coding RNA (LncRNA) in 1991 [35], the list of characterized lncRNAs has grown, with one report suggesting there may be as many as 58,648 lncRNAs in the human transcriptome, many of these not yet annotated [36]. That number is more than twice the total number of annotated ncRNAs currently listed (GRCH38.p13, Ensembl! Release, November 2022). LncRNAs are generally greater than 200 nucleotides (Figure 1), and act as scaffolds, guides, or decoys for proteins and other nucleic acids, modulating gene expression, influencing cellular behavior, and participating in diverse cellular processes, including X-chromosome inactivation. Importantly, lncRNAs expression is cell-, tissue- and even species-specific forms, and, in some cases, lncRNAs are differentially spliced to give the different isoforms more specificity.

Four articles focused specifically on LncRNAs in this Special Issue, with three examining the lncRNA signatures in specific conditions, and one, which was a paper from my group, examining the SNHG14 lncRNA [37]. The *SNHG14* locus is found on chromosome 15q, and often fully or partially deleted by Prader–Willi Syndrome (PWS). The transcript was originally annotated over 20 years ago by Cavaille and colleagues [38], and while it is highly expressed in neuronal tissues, thyroid, as well as multiple cancer types, [37], we still do not completely understand its normal functional role. One interesting and unique feature of the *SNHG14* gene locus is that it is part of a maternally imprinted region of chromosome 15, with only paternal deletions or maternal disomy in this region leading to loss of expression for the full length SNHG14 lncRNA, and, as a result, PWS. In our article, we provide an overview of the gene, its multiple splice variants, including the production of small nuclear ncRNAs, SNORD115 and SNORD116, and new data showing some of the expression patterns in adult mouse brain tissue [37].

Consistent with the altered expression of LncRNAs in cancer, the other three lncRNA focused articles in this Special Issue characterize the LncRNA signatures in endometrial cancer [39], colorectal cancer [40], and cancer-associated endothelial cells undergoing the angiogenic switch during carcinogenesis [41]. Both Sun and colleagues [39] and Song and colleagues [40] sorted LncRNAs by their methylation status, on guanine residues (m7G) and adenine residues (m6A). The 7mG modification is most commonly seen on rRNA and the 5′ methyl guanosine cap of mRNA, but Sun and colleagues were able to identify 10 LncRNAs with this modification, and further separate theses into groups associated with higher risk or lower risk for endometrial cancer progression [39]. Similarly, Song and colleagues used transcriptomic and clinical data from 509 patients, to ultimately classify 7 m6A methylated LncRNAs whole expression pattern could be used as prognostic markers of colorectal cancer progression, specifically tumor grade, vascular infiltration, and immune score [40]. Lastly, in this group of LncRNA papers, Mabeta and colleagues reviewed the role of pro- and anti-angiogenic LncRNA expression in the switch to angiogenesis during carcinogenesis of a variety of tumor types [41]. They conclude that the disruption in the balance of pro- versus anti-angiogenic factors, and the subsequent effect on downstream targets enables the progression of an avascular tumor to a more malignant, angiogenic promoting tumor, and that this LncRNA signature is prognostic for several tissue types.

## 5. Circular Non-Coding RNAs

**Figure 3:**
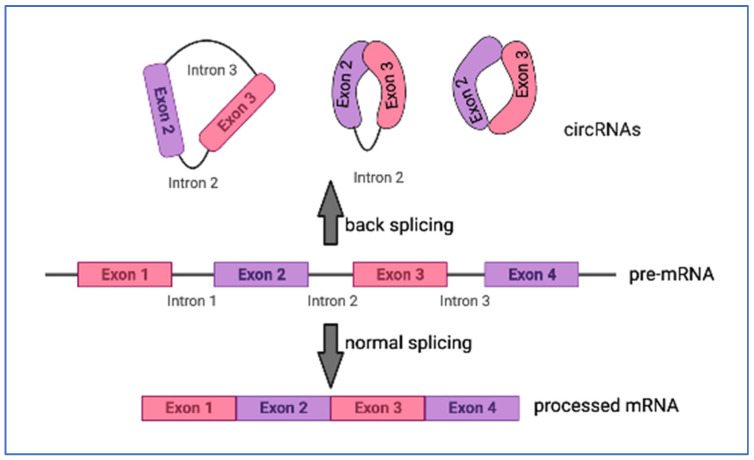
Biogeneration of circRNA. The pre-mRNA can be processed by regular splicing, or in cases where the mRNA is transcribed quickly by RNA pol, and complementary ends are present, and are back-spliced to create circRNAs (Created with BioRender.com, accessed on 25 June 2023).

The first circular non-coding RNA (circRNA) was identified in 1993 as a processed transcript of the testis-determining gene, SRY [42]. It, and now other circRNAs, were shown to be generated by the back-splicing of mRNA transcripts where the 5′ and 3′ splice donor and acceptor sites of an mRNA generated by rapid RNA polymerase II transcription were spliced, and connected to make a circular RNA (Figure 3) [43]. The SRY circRNA consists of a single exon, flanked by introns, and is an miRNA sponge, with no polysomes associated with it in the testicular transcriptome [42]. CircRNAs by their nature are generally greater than 300 nucleotides in length, and, thus, are categorized within the lncRNA family (Figure 1).

In this Special Issue on ncRNAs, two articles focused on circRNA. Zhang and colleagues used liver tissue from a diet-induced obesity mouse model to identify 7469 circRNA within this tissue, as well as 21 that were differentially expressed between the control animals and those with diet-induced obesity [44]. Interestingly, their circRNA species varied in size from less than 200 nucleotides to more than 1200 nucleotides in length, with the most common species (40%) within the 200–400 nucleotide range. Given these data, one may need to re-think the classification of circRNAs within the LncRNA grouping. Furthermore, approximately 80% of these circRNAs had between 1 and 3 exons, whereas significantly fewer extended to the 7–10 exon range [44]. Zhang and colleagues then characterized a circRNA network in their model, focusing on circRNA4842, which is created from the 3rd–5th exon of the *PTEN* tumor suppressor gene. This circRNA is upregulated with high fat diet, and Zhang and colleagues found that mRNA from its parent gene *PTEN* is subsequently downregulated. Translation of this work to humans will be important in identifying the circRNA networks that could be used in the prediction or treatment of obesity.

Jang and colleagues focused on a specific human circRNA, hsa_circ_0003570 that they believe may be useful as a clinical biomarker for the prognosis of hepatocellular carcinoma [45]. Using 121 patients, they showed that higher expression of hsa_circ_0003570 was associated with higher four-year survival rates. The parental gene for hsa_circ_0003570 is *FAM53B*, which is a nuclear protein that works as part of the *Wnt* signaling pathway. No direct role for the Fam53B protein in hepatocellular carcinoma has been characterized to date, but, interestingly, the truncation of the *FAM53B* gene is associated with the development of acute lymphoblastic leukemia [46], and a previous study also linked downregulation of hsa_circ_0003570 with hepatic carcinoma cell lines, and matched tumor and normal tissues from 107 paired biopsies [47].

More than 25,000 circRNAs have been identified in large-scale transcriptomic studies, but many of these are not yet characterized, as indicated by the lower number of total ncRNAs listed in Ensembl! (GRCH38.p13, Ensembl! Release, November 2022). The characterization of the expression patterns of the circRNA, their parent mRNA and their possible mRNA and miRNA targets will lead to future studies that can classify the functionality of these unique members of the ncRNA family.

## 6. The ncRNA-Ome: Studies Examining ncRNA Networks

The next four papers to be discussed focused on ncRNA networks, rather than specific ncRNA types. Song and colleagues describe their findings on the ncRNA network in newly diagnosed Chinese patients with T2D [48]. In total, 24 participants with T2D and 24 healthy controls were included in their study in which RNA was extracted from plasma and used to generate an ncRNA and mRNA transcriptome library. The differentially expressed ncRNAs included 49 miRNAs, and 124 lncRNAs, complemented by 312 mRNAs. Gene ontogeny (GO) and KEGG analysis identified metabolism, genetic processing, fatty acid β-oxidation, and gluconeogenesis as enriched pathways. The group then used STRING network (https://string-db.org, accessed by Song and colleagues, 9 November 2022) to characterize miRNA–lncRNA–mRNA networks in their datasets, leading to the discovery of several ncRNA and mRNAs expression patterns that were correlated with newly diagnosed T2D.

Wang and colleagues were interested in the protein product of mucin gene *MUC14* (officially *EMCN* gene, and endomucin protein) [15] which they predicted to be a potential regulator of breast cancer based on expression level of the MUC family of proteins [49]. Mucin proteins are O-glycosylated proteins that form mucous barriers on epithelial exposed surfaces, including those surfaces in the lungs and breast tissues. Mucin-14/endomucin is a sialoglycoprotein, that has previously been shown to inhibit cell–extracellular matrix interactions [50]. Wang and colleagues demonstrated that Mucin-14 protein levels were significantly downregulated in breast cancer samples, compared to controls, and, together with survival analysis and other pathological associations, went on to suggest that downregulation of Mucin-14 protein was a prognostic for breast cancers. Wang and colleagues then asked if a ncRNA network may also be associated with breast cancers and Mucin-14 protein. They found seven upregulated miRNAs (miR-30a-5p, miR-7-5p, miR-200b-3p, miR-137, miR-200c-3p, miR-30e-5p, and miR-429) in breast cancer patients versus normal breast tissues, with only miR-137, and miR-429 upregulation correlated with poor prognosis. Likewise, in analyzing lncRNA levels, which they predicted would normally act in a tumor-suppressor role in breast cancer, high levels of LINC01128, CCDC18-AS1, SH3BP5-AS1, HOTAIRM1, LINC01140, SGMS1-AS1, LINC01578, or LINC00667 were correlated with better prognosis [49]. Overall, the authors established a Mucin-14/ncRNA network that could have prognostic value for breast cancer diagnosis and staging.

**Figure 4:**
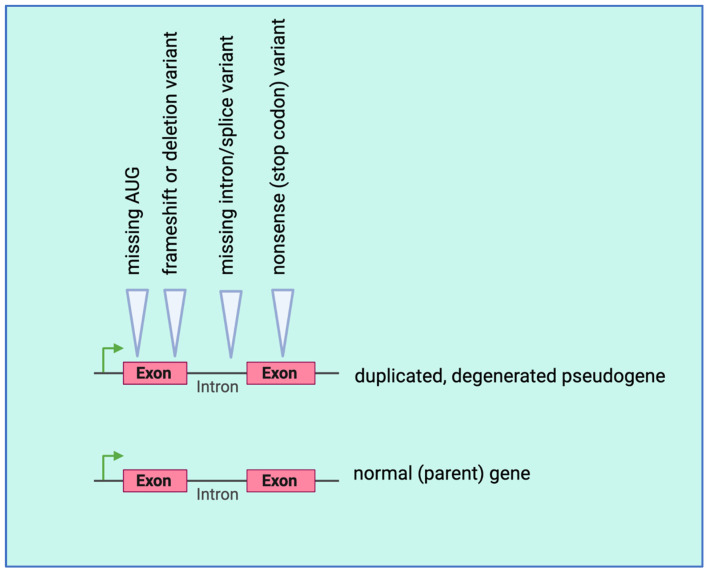
Pseudogenes produce ncRNA transcripts, especially when the promoter is left intact. The pseudogene is derived from a normal, parental gene that became duplicated within our genome. However, due to a lack of some selective pressures on the pseudogene, variants, and deletions can accumulate, rendering the gene non-coding (Created with BioRender.com, accessed on 26 May 2023).

A paper in this Special Issue from Ye and colleagues examined ncRNAs in gastric cancer [51]. They hypothesized that multiple types of ncRNAs form a competitive endogenous RNA (ceRNA) network and reviewed the ceRNA network in gastric cancer. This paper provides a comprehensive table of databases and other resources for ceRNAs that would be a great starting place for individuals thinking about the ceRNA network in their tissue or disease of interest. In the last section of their review, Ye and colleagues discuss the role of pseudogenes as ceRNAs, which directly leads to our last paper to be discussed by Carron and colleagues [52]. This paper reviews the plausible role of pseudogene transcripts, which are classified as ncRNA precisely because they have degenerated to the point where no proteins are made from the duplicated gene transcript, and often negatively regulate the sense RNA from the parental genes (Figure 4). There are likely over 14,000 pseudogenes that were initially considered nonfunctional, but now evidence suggests that some can function as ncRNAs [53]. In addition to reviewing this ncRNA class, Carron and colleagues perform an in silico analysis to detect dysregulated pseudogenes in 219 patients with head and neck cancers. They identify 370 transcripts in this group (compared to controls), and find that the some common type of variant in these pseudogenes (compared to the parent gene) were single nucleotide variants (found in 96.8% of their dataset) followed by deletions and insertions that were found in 1.9% and 1.3% of their dataset, respectively [52]. In a tour-de-force bioinformatic/in silico study, the authors further narrow down the list to 10 pseudogene transcripts with the most prognostic value, discussing how up- and down-regulation of these transcripts may promote head and neck cancers.

## 7. Conclusions

The discovery of ncRNAs has revolutionized our understanding of the complexity of gene regulation and cellular processes. These molecules are involved in a myriad of biological functions, ranging from embryonic development and immune response to cancer progression and neurological disorders, opening new avenues for research and potential therapeutic applications, as they represent a vast unexplored landscape of molecular mechanisms and targets.

We are only beginning to understand the role of differential regulation or function of ncRNAs caused by genetic and epigenetic perturbations, such as single nucleotide variants (SNV), deletions, insertions, and histone/DNA modifications. Emerging roles for ncRNAs have been identified in neurological, cardiovascular, immune, and digestive systems, to name a few. It is now time to explore how altered function or expression of ncRNAs can lead to disease. Examples include cancer, Prader–Willi Syndrome, cardiac arrhythmias, and spinal motor neuron disease, to name a few. As we continue to understand and further annotate this class of RNAs, articles from this Special Issue have provided specific examples of ncRNAs involved in human health and diseases, as well as strategies to target ncRNAs signatures that could lead to improved therapeutic identification, and interventions for some conditions, especially cancer.
